# Axillary treatment for patients with early breast cancer and lymph node metastasis: systematic review protocol

**DOI:** 10.1186/1477-7819-11-6

**Published:** 2013-01-14

**Authors:** Amit Goyal, Lelia Duley, Apostolos Fakis

**Affiliations:** 1 Department of Surgery, Royal Derby Hospital, Derby, England; 2 Nottingham Clinical Trials Unit, University of Nottingham, Nottingham, England; 3 Research & Development Department, Royal Derby Hospital, Derby, England

## Abstract

**Background:**

For patients with early breast cancer and lymph node metastasis, axillary treatment is widely recommended. This is either surgical removal of the axillary lymph nodes, or axillary radiotherapy. The rationale for axillary treatment is that it will reduce the risk of recurrence in the axilla, and may improve survival. However, both treatments are associated with adverse effects, such as lymphedema, pain and sensory loss, and are costly to the health services and to patients. With improvements in adjuvant therapy, routine axillary treatment may no longer offer any overall advantage.

**Objectives:**

To assess the short and long term benefits and adverse effects of routine axillary treatment (axillary lymph node clearance or axillary radiotherapy) for patients with lymph node positive early-stage breast cancer.

**Methods/Design:**

Criteria for potentially eligibility for the study will be that the participants are men and women with early breast cancer and lymph nodes with metastasis. The study compares either axillary treatment with no axillary treatment, or axillary node clearance with axillary radiotherapy, and the study is a randomized trial. Primary outcomes are axillary recurrence, disease-free and overall survival. Secondary outcomes include breast or chest wall recurrence, distant metastasis, time to axillary recurrence, axillary recurrence-free survival, arm morbidity, quality of life and health economic costs. The search strategy will include the Cochrane Central Register of Controlled Trials, MEDLINE, EMBASE and WHO International Clinical Trials Registry Platform (ICTRP) search portal. Two independent reviewers will assess studies for inclusion in the review, assess study quality and extract data. Characteristics of included studies will be described. Meta-analysis will be conducted using ReVman software.

**Comment:**

This review addresses an important clinical question, and results will inform clinical practice and health care policy.

## Background

### Description of the condition

Breast cancer is the most frequent cancer affecting women worldwide [1]. For example, in the UK more than 45,000 people are diagnosed with breast cancer each year and the majority (80%) of these patients undergo surgical treatment [2]. Patients with early-stage invasive breast cancer undergo breast surgery, which could be lumpectomy (wide local excision) or mastectomy. These patients also have one or two lymph nodes (glands) removed from the axilla (armpit) during this surgery to check if the cancer has spread to the nodes; a procedure called sentinel node biopsy (SNB). In around a quarter of patients, the cancer has spread to the nodes. Current practice is that these patients with cancer in the nodes undergo axillary treatment, which is either surgical removal of the remaining axillary nodes or axillary radiotherapy.

### Description of the intervention

Axillary node clearance (ANC) is removal of all axillary nodes in the armpit in patients found to have cancer spread to lymph nodes (sentinel nodes) removed during sentinel node biopsy. This is usually performed at a second operation, which can be difficult due to scarring from the first operation (sentinel node biopsy). A drain is left in the armpit for a few days afterwards [3]. The operation lasts one to two hours and requires a stay in hospital of up to five days. It delays the patient’s return to day-to-day activities and paid work [4].

Axillary radiotherapy (ART) is radiation treatment of the remaining axillary nodes. It is used instead of axillary node clearance for some patients. The prescribed radiation dose is given on a daily basis, five days a week for three to five weeks. Axillary radiotherapy is offered in some specialist centres, and patients may need to travel a considerable distance for treatment.

### How the intervention might work

Axillary treatment can eliminate residual disease in the axilla, decrease axillary recurrence and, perhaps, improve overall survival by improving local control. One breast cancer death out of four local recurrences can be prevented over the 15 year period [5]. ANC provides information on the number of positive nodes (extent of nodal involvement) and this may influence adjuvant systemic therapy and radiotherapy decisions.

Axillary recurrence rates following ANC or ART have been reported to be as low as 1 or 2%, however, both are associated with significant long term problems such as pain, arm swelling (lymphedema), restricted shoulder movement, and sensory changes in the arm and hand (for example, numbness) [4,6-8].

### Why it is important to perform this review

The value of axillary treatment in the era of early detection, small tumors and adjuvant systemic therapy may be more limited than in the past. Less than 50% of patients with sentinel node metastases are found to have additional nodal disease at the second operation, ANC [9]. Once axillary node metastases are defined by information for systemic adjuvant therapy is adequate without the need for harvesting more nodes, since systemic therapies are not usually governed by the number of node metastases [10]. Most patients receive adjuvant systemic therapy and we now have molecular determinants of prognosis and predictors of treatment benefit. Moreover, the lower axilla is treated inadvertently in all patients as it is included in the irradiation field during whole breast radiotherapy, or some lower level axillary nodes are included in the mastectomy specimen [11].

Physicians are at a crossroad in terms of effective axillary treatment with the Z0011 trial [12] suggesting a ‘de-escalation’ of nodal therapy, but there are some concerns about this approach as suggested by the MA20 trial [13]. This has led us to review existing evidence to address the prevailing uncertainty, as the harms of axillary treatment may outweigh the benefits if the risk of axillary recurrence is low.

## Objectives

To assess the short and long term benefits and adverse effects of axillary treatment (axillary lymph node clearance or axillary radiotherapy) for patients with lymph node positive early-stage breast cancer.

## Methods

### Criteria for considering studies for this review

#### Types of studies

Randomized and quasi-randomized clinical trials evaluating: 1) Axillary treatment (axillary lymph node clearance or axillary radiotherapy) versus no axillary treatment; 2) Axillary lymph node clearance versus axillary radiotherapy.

#### Types of participants

Patients with clinically node negative early-stage invasive breast cancer and positive axillary lymph nodes on axillary dissection, axillary sampling or sentinel node biopsy, regardless of primary treatment for breast cancer. Both mastectomy and breast conservation patients will be included. Early breast cancer includes tumors classified as AJCC stage I to IIIA. We will exclude women who have had previous surgical treatment for the current tumor and those with a history of breast cancer. We will not exclude patients based on age, race or histological type.

#### Types of intervention

Axillary treatment (axillary lymph node clearance or axillary radiotherapy).

#### Types of outcome measures

In this review, where possible, we will extract data at 5, 10, 15, 20 and 25 years.

#### Primary outcomes

1. Axillary *(Regional)* recurrence: defined as tumor recurrence in lymph nodes draining the primary tumor site, namely, nodes in the ipsilateral axilla, infraclavicular fossa, supraclavicular fossa and interpectoral area.

2. Disease-free survival: defined as the interval between the date of breast cancer diagnosis and the date of the first loco-regional or systemic recurrence, or date of death, whichever comes first.

3. Overall survival: defined as the interval from the date of breast cancer diagnosis until the date of death from any cause.

#### Secondary outcomes

1. Breast/chest wall *(Local)* recurrence: this includes recurrence after mastectomy in the skin or soft tissue of the chest wall, or within the treated breast following breast conservation.

2. Distant metastasis: all other sites of recurrence are included under this heading and are classified as: soft-tissue category, visceral category, central nervous system and skeletal spread.

3. Time to axillary recurrence: the time between the date of breast cancer diagnosis and the date of first sign of axillary recurrence.

4. Axillary recurrence-free survival: the time interval between the date of diagnosis and the date of first sign of axillary recurrence without evidence of distant disease, or date of death, whichever comes first.

5. Arm morbidity: which includes lymphedema, shoulder stiffness, paresthesia, pain, loss of functional capacity, winging of scapula and wound contracture or scarring problems. Where any grading systems are used for the severity of these complications, scores will be recorded.

6. Quality of life (using trial-specific instruments).

7. Health economic costs.

### Search methods for identification of studies

#### Electronic searches

We will search the following databases.

(a) The Cochrane Central Register of Controlled Trials (CENTRAL) (*The Cochrane Library,* current issue). See Appendix 1.

(b) MEDLINE (via OVID) (from January 1980 until the search date). See Appendix 2.

(c) EMBASE (via OVID) (from January 1980 until the search date). See Appendix 3.

(d) The WHO International Clinical Trials Registry Platform (ICTRP) search portal (http://apps.who.int/trialsearch/) for all prospectively registered and ongoing trials. See Appendix 4.

We will not apply search restrictions to age, race, tumor size or histological type. We will not impose any language restrictions.

### Searching other resources

#### References from published studies

We will check reference lists from eligible trials selected by electronic searching to identify further relevant trials.

#### Conference proceedings

We will search the American Society of Clinical Oncology and San Antonio Breast Cancer Symposium conference proceedings.

#### Unpublished literature (electronic)

We will search the UK Clinical Trials Gateway (UKCTG) and the National Institute for Health Research Clinical Research Network (NIHR CRN) Portfolio database for details of ongoing trials in the UK.

#### Personal communication

We will contact, by e-mail, the corresponding authors for missing data that are needed for the systematic review.

### Data collection and analysis

#### Selection of studies

Two authors will independently scan the title, abstract and keywords of every record identified by the search. We will assess the full articles if the information given suggests that the study may conform to our criteria. We will resolve differences in assessment by discussion and, in cases of disagreement, we will consult another review author.

#### Data extraction and management

Two authors will perform data extraction independently using a standard form, and we will resolve disagreements by discussion. We will enter data on outcome measures into Review Manager 5.1 (RevMan 2011) software for analysis. Where possible, we will extract data on tumor and patient characteristics, size of nodal metastasis, surgery performed and adjuvant treatments.

#### Assessment of risk of bias in included studies

Two review authors will independently assess the quality and risk of bias of the eligible studies using the Cochrane Collaboration’s risk of bias tool [14]. Any disagreements will be resolved by discussion or by involving a third assessor.

##### Sequence generation (checking for possible selection bias)

We will describe for each included study, the methods used to generate the allocation sequence. The methods will be assessed as:

• low risk of bias (any truly random process, for example, random number table; computer random number generator),

• high risk of bias (any non-random process, for example, odd or even date of birth; hospital or clinic record number) or,

• unclear risk of bias.

##### Allocation concealment (checking for possible selection bias)

We will assess whether intervention allocation could have been foreseen in advance of, or during recruitment, or changed after recruitment:

• low risk of bias (for example, telephone or central randomization; consecutively numbered, sealed opaque envelopes);

• high risk of bias (open random allocation; unsealed or non-opaque envelopes, alternation; date of birth) or,

• unclear risk of bias.

##### Blinding (checking for possible performance bias)

Given the nature of the interventions being evaluated, blinding of either the care providers or the patients receiving care was not feasible. We will assess methods used to blind outcome assessment as:

• low, high or unclear risk of bias for participants;

• low, high or unclear risk of bias for personnel.

##### Incomplete outcome data (checking for possible attrition bias through withdrawals, dropouts, protocol deviations)

We will indicate for each included study, the completeness of outcome data for each main outcome, including attrition and exclusions from the analysis. We will state the number lost to follow-up (compared with the total randomized participants), reasons for attrition/exclusion where reported, and any re-inclusions in analyses which we undertake.

We will assess methods as:

• low risk of bias (for example, no missing outcome data; missing outcome data balanced across groups);

• high risk of bias (for example, numbers or reasons for missing data imbalanced across groups; ‘as treated’ analysis done with substantial departure of intervention received from that assigned at randomization) or,

• unclear risk of bias.

##### Selective reporting bias and other sources of bias

We will describe for each included study how we investigated the possibility of selective outcome reporting bias and what we found. We will assess the methods as:

• low risk of bias (where it is clear that all of the study’s pre-specified outcomes and all expected outcomes of interest to the review have been reported);

• high risk of bias (where not all the study’s pre-specified outcomes have been reported; one or more reported primary outcomes were not pre-specified; outcomes of interest are reported incompletely and so cannot be used; study fails to include results of a key outcome that would have been expected to have been reported) or,

• unclear risk of bias.

##### Other bias

We will describe for each included study, any important concerns we have about other possible sources of bias. We will assess whether each study is free of other problems that could put it at risk of bias:

• low risk of other bias;

• high risk of other bias or,

• unclear whether there is risk of other bias.

##### Overall risk of bias

We will make explicit judgements about risk of bias for important outcomes both within and across studies. With reference to (1) to (6) above, we will assess the likely magnitude and direction of the bias and whether we consider it likely to impact on the findings. We plan to explore the impact of the level of bias through undertaking sensitivity analyses; temporarily removing those studies at high risk of bias from the meta-analysis to see what impact this will have on the treatment effect.

### Measures of treatment effect

We will carry out statistical analysis using Review Manager 5.1 (RevMan 2011). We will use fixed-effect meta-analysis for combining data in the absence of heterogeneity. For those outcomes where there are moderate or high levels of heterogeneity, where clinically meaningful, we will use random-effects analysis and these results will be presented as average treatment effects.

For dichotomous data, we will present results as summary risk ratio (RR) with 95% confidence intervals. For continuous data, we will use the mean difference if outcomes were measured in the same way between trials. We will use the standardized mean difference to combine trials that measured the same outcome, but using different methods. If there is evidence in the trials of abnormally distributed data, we will report this.

### Unit of analysis issues

We anticipate that we will only find trials in which the unit of randomization was the individual patient. However, if we find cluster randomization trials they will be included.

### Dealing with missing data

For included studies, we will note levels of attrition in the risk of bias tables. We plan to explore the impact of including studies with high levels of missing data in the overall assessment of treatment effect by using sensitivity analysis. Where possible we will analyse all cases according to randomization group, irrespective of whether or not study participants received the intended intervention.

### Assessment of heterogeneity

We will examine heterogeneity between the trials by visually examining the forest plots to judge whether there are any apparent differences in the direction or size of the treatment effect between studies. We will also consider the I-squared and T-squared statistics and the *P-*value of the Chi-squared test for heterogeneity. If we identify heterogeneity among the trials (if the value of I-squared is greater than 30%, and the value of T-squared is greater than zero or the *P-*value of the Chi-squared test for heterogeneity is greater than 0.1), we will explore it by pre-specified subgroup analysis and by performing sensitivity analysis.

### Assessment of reporting biases

We will not formally assess reporting bias; without access to study protocols it is difficult to know whether or not there has been outcome-reporting bias. However, we will note where we have any concerns about reporting bias (for example, where key outcomes do not seem to be reported).

### Subgroup analysis and investigation of heterogeneity

If we identify substantial heterogeneity, we will investigate it using subgroup analyses and sensitivity analyses. We will consider whether an overall summary is meaningful, and if it is, we will use random-effects analysis to produce it. We plan to carry out the following subgroup analyses:

1. Type of axillary treatment allocated at trial entry: axillary lymph node clearance, axillary radiotherapy; unknown or mixed.

2. Size of nodal metastasis in the sentinel node biopsy: macrometastases (tumor deposit greater than 2.0 mm in the largest dimension), micrometastases (greater than 0.2 mm and/or more than 200 cells in a single histological cross-section, but none greater than 2 mm), isolated tumor cells (small cluster of cells not greater than 0.2 mm, or single tumor cells, or a cluster of fewer than 200 cells in a single histological cross-section), unknown or mixed.

3. Type of primary breast surgery: mastectomy, lumpectomy, mixed or unknown.

4. Subgroup analyses will be restricted to the primary outcomes.

For fixed-effect meta-analysis, we will carry out an interaction test to examine subgroup differences. For both fixed- and random-effects meta-analysis, we will examine the confidence intervals for subgroups; with overlapping confidence intervals potentially suggesting no important differences between subgroups.

### Sensitivity analysis

We plan to include a sensitivity analysis based on temporarily excluding trials that were not of high quality. If this exclusion leads to a substantive difference in the overall results, we will exclude quasi-random studies or those with serious attrition. Only the primary outcomes will be included in the sensitivity analysis.

## Appendix 1

### Search strategy: CENTRAL

#1. (breast cancer): ti,ab,kw in Trials

#2. MeSH descriptor **Breast Neoplasms** explode all trees

#3. (#1 or #2)

#4. (four node sampling): ti,ab,kw or (4 node sampling): ti,ab,kw in Trials

#5. (axillary node dissection): ti,ab,kw or (axillary node clearance): ti,ab,kw in Trials

#6. MeSH descriptor **Lymph Node Excision** explode all trees

#7. MeSH descriptor **Axilla** explode all trees

#8. (#6 or #7)

#9. (#4 or #5 or #8)

#10. MeSH descriptor **Radiotherapy** explode all trees

#11. (#7 and #10)

#12. (#3 and #9 and #11)

#13. (randomised controlled trial):pt or (randomized controlled trial): pt or (RCT): pt or (randomised controlled trial): ti,ab,kw or (randomized controlled trial): ti,ab,kw in Trials

#14. (#12 and #13)

## Appendix 2

### Search strategy: MEDLINE (OVID) 1980 – date of search

1. breast cancer.mp. or exp Breast Neoplasms/

2. four node sampling.mp.

3. 4 node sampling. ab,kw,ti.

4. axillary node dissection.mp.

5. axillary node clearance.mp.

6. exp Lymph Node Excision/

7. exp Axilla/

8. 6 and 7

9. 2 or 3 or 4 or 5 or 8

10. axillary radiotherapy.mp.

11. exp Radiotherapy/

12. 7 and 11

13. 10 or 12

14. randomised controlled trial.mp,pt.

15. randomized controlled trial.mp,pt.

16. RCT.mp,pt.

17. 14 or 15 or 16

18. 1 and 9 and 13 and 17

19. limit 18 to yr="1980 -Current"

## Appendix 3

### Search strategy: EMBASE (OVID) 1980 - date of search

1. breast cancer.mp. or exp breast cancer/

2. four node sampling.mp.

3. 4 node sampling.mp.

4. axillary node dissection.mp.

5. axillary node clearance.mp.

6. exp lymphadenectomy/

7. exp axilla/

8. 6 and 7

9. 2 or 3 or 4 or 5 or 8

10. axillary radiotherapy.mp.

11. exp radiotherapy/

12. 7 and 11

13. 10 or 12

14. randomised controlled trial.mp,pt.

15. randomized controlled trial.mp,pt.

16. RCT.mp,pt.

17. 14 or 15 or 16

18. 1 and 9 and 13 and 17

## Appendix 4

### Search strategy: WHO ICTRP

Title: Breast cancer and axillary

AND

Condition: Breast cancer or breast neoplasm

AND

Intervention: Four node sampling or axillary dissection or axillary clearance or axillary radiotherapy

Recruitment Status: All

Date of Registration: 01/01/1980 – search date.

## Competing interests

The authors are investigators on the proposed POSNOC (POsitive Sentinel Node: adjuvant therapy alone versus adjuvant therapy plus Clearance or axillary radiotherapy: A randomized controlled trial of axillary treatment in women with early stage breast cancer who have metastases in one or two sentinel nodes) trial.

## Authors’ contributions

All three authors have contributed to drafting the protocol. All authors read and approved the final manuscript.
